# The Effect of Wearable-Based Real-Time Feedback on Running Injuries and Running Performance: A Randomized Controlled Trial

**DOI:** 10.1177/03635465231222464

**Published:** 2024-01-29

**Authors:** Bas Van Hooren, Guy Plasqui, Kenneth Meijer

**Affiliations:** † NUTRIM School of Nutrition and Translational Research in Metabolism, Maastricht University Medical Centre+, Department of Nutrition and Movement Sciences, Maastricht, the Netherlands; Investigation performed at the School of Nutrition and Translational Research in Metabolism, the Department of Nutrition and Movement Sciences, Maastricht, the Netherlands

**Keywords:** biofeedback, biomechanics, motivation, technology, wearables; running

## Abstract

**Background:**

Running technique and running speed are considered important risk factors for running injuries. Real-time feedback on running technique and running speed by wearables may help reduce injury risk.

**Purpose:**

To investigate whether real-time feedback on spatiotemporal metrics and relative speed by commercially available pressure-sensitive insoles would reduce running injuries and improve running performance compared with no real-time feedback.

**Study Design:**

Randomized controlled trial; Level of evidence, 1.

**Methods:**

A total of 220 recreational runners were randomly assigned into the intervention and control groups. Both groups received pressure-sensitive insoles, but only the intervention group received real-time feedback on spatiotemporal metrics and relative speed. The feedback aimed to reduce loading on the joint/segment estimated to exhibit the highest load. Injury rates were compared between the groups using Cox regressions. Secondary outcomes compared included injury severity, the proportion of runners with multiple injuries, changes in self-reported personal best times and motivation (Behavioral Regulation in Exercise Questionnaire–2), and interest in continuing wearable use after study completion.

**Results:**

A total of 160 participants (73%) were included in analyses of the primary outcome. Intention-to-treat analysis showed no significant difference in injury rate between the groups (Hazard ratio [HR], 1.11; *P =* .70). This was expected, as 53 of 160 (33%) participants ended up in the unassigned group because they used incorrect wearable settings, nullifying any interventional effects. As-treated analysis showed a significantly lower injury rate among participants receiving real-time feedback (HR, 0.53; *P =* .03). Similarly, the first-time injury severity was significantly lower (–0.43; *P =* .042). Per-protocol analysis showed no significant differences in injury rates, but the direction favored the intervention group (HR, 0.67; *P =* .30). There were no significant differences in the proportion of patients with multiple injuries (HR, 0.82; *P =* .40) or changes in running performance (3.07%; *P =* .26) and motivation. Also, ~60% of the participants who completed the study showed interest in continuing wearable use.

**Conclusion:**

Real-time feedback on spatiotemporal metrics and relative speed provided by commercially available instrumented insoles may reduce the rate and severity of injuries in recreational runners. Feedback did not influence running performance and exercise motivation.

**Registration:**

NL8472 (Dutch Trial Register).

Running is one of the most popular sporting activities and has the strongest evidence for health benefits compared with other sports.^50^ Running is also characterized by a high dropout, with dropout rates of up to 50% reported during running intervention programs. Running-related injuries are the most common reason for dropout,^26^ with running technique and inappropriate progression of training load (ie, too much, too soon, too fast) being considered important risk factors for running-related injuries.

A large number of studies have used real-time feedback to modify running techniques in an attempt to reduce risk factors for running injuries. Although several studies have been able to effectively modify risk factors of running injuries^45,46,49^ or reduce actual running-related injuries,^10^ most of these studies were performed in a laboratory environment, where expensive motion capture equipment was used to analyze and provide real-time feedback on running technique. Moreover, participants were required to visit the laboratory multiple times for technique retraining. These requirements reduce the applicability of the findings, as most runners do not have access to this equipment and do not have the time to visit a laboratory multiple times. Furthermore, the modified running technique observed in a laboratory may not fully translate to outdoor running^70^ and may also partly return to the baseline without periodic gait retraining,^11,49,68^ both of which reduce the effectiveness of laboratory-based interventions. Another limitation of laboratory-based studies is that they typically provide feedback only on running technique and provide no guidance on an appropriate training load to prevent running injuries and optimize performance. However, real-time feedback on running technique alone is likely suboptimal compared with an integrated approach whereby guidance is also provided on training load. While training load reflects the combination of volume, intensity, and frequency of training, intensity reflects a modifiable and potentially relevant factor for real-time feedback. In support of the potential relevance of intensity-related feedback, large-scale wearable data suggest that recreational runners often perform training sessions at a relatively higher intensity compared with well-trained runners,^2^ which may, in turn, partly contribute to their higher injury risk by exacerbating fatigue^7^ and increasing tissue damage and the corresponding probability of failure (ie, injury).^19,24^

Wearables offer a promising method to quantify running technique and training intensity outside of the laboratory, and they can provide real-time feedback on these aspects.^64^ This feedback may, in turn, reduce injury rates and enhance running performance. In the single randomized controlled trial that investigated the effects of real-time feedback on running injuries in an in-field setting performed to date, Morris et al^44^ found no overall effect of real-time feedback that aimed to modify a rearfoot strike to a nonrearfoot strike on the incidence of running injuries. This may be related to the following factors: the biomechanical outcome chosen to use in feedback (ie, only footstrike modification); the rate at which footstrike was changed; the absence of feedback on other biomechanical outcomes or relative intensity; low (14%) compliance that in turn may have resulted from the requirement for participants to attach an accelerometer to the tibia during each run; inability to choose their running pace freely; and having to wear earphones to receive real-time feedback.

Instrumented insoles that measure running technique and speed while providing real-time feedback via a mobile phone may overcome most of the limitations of previous studies. Specifically, the wireless and lightweight nature of recently developed pressure insoles allows them to be used in-field, and, in contrast to other insoles, makes them less likely to interfere with running technique.^33^ Moreover, insoles are likely easier to use than other wearables, such as tibial-mounted accelerometers, thereby improving compliance. Pressure-sensitive insoles can accurately measure spatiotemporal running metrics during various running conditions.^66^ These metrics can be used to determine the relative load on the different joints/segments based on correlations between spatiotemporal metrics and tissue loading reported in the literature.^5^ Moreover, real-time feedback on spatiotemporal metrics may also benefit the running economy for those whose self-selected technique deviates from their most economical technique. Specifically, novice runners often adopt a too-low step frequency relative to their theoretically most economical step frequency^65^; and increases in step frequency may, therefore, improve running economy for these runners.^43,65^ Finally, running speed derived from the Global Positioning System (GPS) or an accelerometer embedded in the instrumented insoles can provide real-time feedback on the relative running speed.

The effectiveness of wearables to reduce injuries and improve running performance remains unknown. Therefore, the primary aim of this study was to investigate whether real-time feedback that aims to alter relative loading and relative speed by providing feedback on spatiotemporal metrics and speed using commercially available pressure-sensitive insoles is effective at reducing running injuries compared with no real-time feedback. We hypothesized that the feedback provided by such a wearable would lead to lower injury rates and severity as compared with no real-time feedback. Finally, a low motivation to run has been suggested to contribute to dropout.^64^ A second way by which wearables may contribute to lower dropout rates is by improving motivation to exercise; for example, by increasing perceived competence by means of real-time feedback. Thus, a secondary aim was to investigate whether real-time feedback is also more effective at improving running performance and motivation to exercise.

## Methods

### Study Registration

This randomized controlled trial was registered at the Dutch Trial Register (ID No. NL8472), approved by the local ethics committee (No. NL72989.068.20), conducted according to the declaration of Helsinki, and reported following the CONSORT (Consolidated Standards of Reporting Trials)^42^ and CERT (Consensus on Exercise Reporting Template)^59^ guidelines. All participants signed an informed consent form before the measurements.

### Sample Size Determination

The sample size was estimated a priori for a Cox regression using an online tool with an alpha level of .05 and a power of 0.80 (https://sample-size.net/sample-size-survival-analysis/). We assumed a mean injury rate of 0.29 in the control group based on the lowest injury rate reported in several cohort studies^10,31,44^ and an injury rate ratio of 0.55 between groups over a 6-month period.^10,40^ Also, we increased the sample size by 30% to account for dropout and low compliance,^10^ resulting in a total sample size of 220 runners (110 intervention; 110 control group). This sample size is roughly comparable with other randomized controlled trials that investigated the effects of real-time technique feedback on injuries, with sample sizes ranging from 191 to 390 participants.^10,44^

### Participants

Healthy recreational runners were recruited between December 2020 and April 2021 using social media platforms, flyers, and online advertisements at popular Dutch running competitions and running shoe stores, and via athletics federations. The inclusion and exclusion criteria are described in detail in the Appendix (available in the online version of this article).

### Inclusion and Exclusion Criteria

The inclusion criteria were as follows: (1) self-assessed recreational runners who were running a minimum twice per week, with a minimum total distance of 10 km per week and a maximum of 45 km per week at the time of inclusion; (2) age between 18 and 65 years; (3) proficient in the English language; and (4) interest in training toward being able to run a distance between 10 km and half marathon. The exclusion criteria were as follows: (1) no email address or internet access; (2) smartphone that was not suitable for real-time feedback (eg, older operating system); (3) participation in other sports for >3 hours per week; (4) major or minor lower extremity injury in the past 6 or 3 months, respectively; (5) contraindications for vigorous physical activity, such as pregnancy or having been pregnant in the previous 6 months, discomfort during running, and cardiovascular, metabolic, or pulmonary adverse health conditions—for example, stroke, heart disease, pain in the chest, diabetes, and chronic obstructive pulmonary disease; (6) a body mass index of >27.5; and (7) participation in trail running more than once a week.

### Patient Involvement

No patients were involved in the design of this study.

### Equity, Diversity, and Inclusion Statement

Our baseline study sample included an equal number of men and women from various age groups and demographic backgrounds to accurately represent the typical recreational running sample. We did not do subgroup analysis for age or sex because of the small sample size. Our author team had a diverse expertise and included 1 early career (<7 years of research experience) and 2 midcareer (15-25 years of experience) researchers. Moreover, all authors ranged in their own experience with running from novice to elite levels.

### Randomization and Blinding

After completing the informed consent form, each participant was assigned a number that had to be specified in the beta application created for the study to ensure the anonymity of the collected data for the remaining study. Participants were randomized into either a real-time feedback group or a control group, with group assignment determined using an online research randomizer (ResearchRandomizer.org) by 1 of the researchers (B.V.H.). Stratification was done based on sex. Because of logistical reasons, it was not possible to blind the researcher who performed the data analysis (B.V.H.) to the group allocation.

### Equipment

All participants were provided with pressure-sensitive insoles (ARION; ATO-Gear) that could connect with an application each participant installed on his or her mobile phone. The insoles could gather data up to 5 to 6 hours consecutively and could quantify running technique during prolonged running sessions. Most participants received a 3-dimensional printed prototype device that was subsequently developed into a commercial product (see Appendix for more details, available online).

### Baseline Run

After receiving instructions on using the wearable, all participants performed a baseline run at a comfortable speed—that is, they were instructed to run at a speed where they could talk comfortably (Appendix, available online)—with a duration of a representative training distance. During this run, the wearable assessed their baseline spatiotemporal metrics, and the mean speed was taken as the comfortable running speed.

### Intervention

Most runners nowadays run with a wearable.^30,52^ For example, 86% of the runners preparing for the 2014 Eindhoven half marathon ran with a wearable monitoring device.^30^ Running wearables typically provide real-time information on GPS-derived metrics such as running distance, duration, and absolute running speed.^12^ To mimic the information that runners obtain with wearables typically used in practice, both the intervention and the control groups were provided with real-time updates on their running distance, duration, and absolute running speed. Moreover, participants in both groups could also review a summary of the recorded biomechanical metrics (eg, cadence, contact time) after each training session in the application. This summary showed only the mean value during the run and did not contain any information on how to use the summary information for injury prevention or performance enhancement purposes. We chose to provide participants in both groups with a summary of the recorded biomechanical metrics after the run as these metrics are also often available when running with other wearables, such as a sports watch.^1,64^ Participants in the intervention group were additionally provided real-time feedback on spatiotemporal parameters and relative speed during their run. This real-time feedback also provided specific instructions on what to do; for example, “try to increase your cadence.” The control group could not see any real-time details on spatiotemporal metrics and relative speed in the application. The primary difference between groups was therefore the provision of real-time feedback with specific instructions on what to change/do in the intervention group and the absence of real-time feedback in the control group. The intervention was intended to last a minimum of 6 months with a maximum of 1 year. This variable duration was allowed because a significant proportion of the participants experienced issues with installation of the beta application, and the runners’ involvement in the study did therefore not begin at the same time.

### General Training Guidelines

All participants were provided with general training guidelines as detailed in the Appendix (available online) in an attempt to balance training between the groups.

### Real-Time Feedback

**Figure 1.**
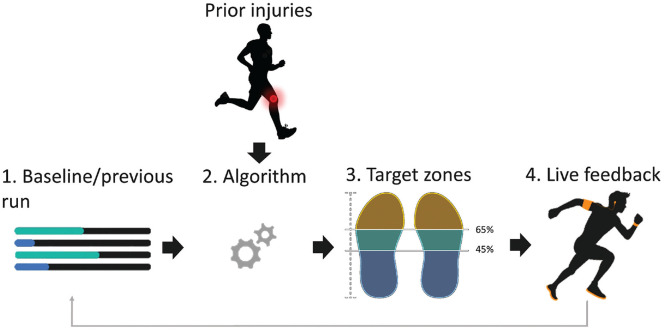
Flowchart of the wearable process for generating feedback. (1) First, the participants’ cadence, footstrike, and comfortable running speed were determined during a “standardized” baseline run using the instrumented insoles and the Global Positioning System (GPS) of the phone, respectively. (2) The parameters measured during this baseline run were used as input to an algorithm that determined the goal for the next session (ie, cadence, footstrike, or running speed), (3) along with the target zones. (4) Real-time feedback was provided when the participant deviated from the target zone. Previous/current injuries could also be specified in the application, and this influenced the feedback provided by the algorithm.

The wearable used data from the pressure sensors (150 Hz), inertial measurement unit (30-50 Hz), and GPS to compute various spatiotemporal metrics such as cadence and footstrike index. These metrics were used as inputs to an algorithm that used correlations reported in the literature (eg, Barton et al^5^) to infer the relative loading of the following two body segments: foot/ankle/lower leg or knee/upper leg. For example, a relatively low step frequency combined with a very pronounced heel strike was assumed to result in a relatively higher load at the knee than at the Achilles tendon or foot.^5^ This load was inferred from spatiotemporal metrics as measured during the baseline run and subsequent runs, and was used with information about previous injuries as input to an algorithm to generate individual target zones for real-time feedback (Figure 1). This algorithm attempted to gradually reduce the loading of the body part with the highest estimated load by providing real-time feedback on modifiable spatiotemporal metrics via a smartphone to reduce injury risk.

Modifications in the relative load and speed were achieved by determining target zones for running speed, cadence, and footstrike index and providing feedback on these metrics. These 3 metrics were chosen as variables for real-time feedback because they were considered easy modifiable by runners and because changes in these metrics would likely affect tissue/joint loading. For example, if the load at the knee was estimated to be high relative to the other body parts based on a low cadence and pronounced rearfoot strike, the feedback instructed the runner to increase cadence, which in turn was expected to reduce knee loading.^5^ The focus for a particular session was prioritized according to a hierarchy with cadence selected ahead of footstrike, and intensity sessions recommended only after successfully completing sufficient technique sessions. When there was no current injury or pain, the wearable used the relative loading inferred from spatiotemporal metrics and the literature to determine a target zone with the goal of gradually reducing the loading of the body part with the highest load. When a current injury/discomfort was specified in the application, the wearable attempted to reduce loading on this body segment specifically. As a secondary goal, the wearable also aimed to improve running economy through biomechanical alterations. Real-time feedback was provided when the participant deviated from the target zone. Specifically, feedback was provided via a mobile phone using auditive instructions (eg, “try to reduce your step frequency” or “try to run a little faster”) when the moving mean over the past 20 steps was 5% lower or higher than the target value. Auditive feedback was provided again after another 20 steps if the moving mean was still 5% higher or lower than the target value. Visual self-determined feedback was also available on the mobile phone for participants in the intervention group. The participants could wear the phone in their hands or in an arm pocket during their runs and combine this with headphones if desired. Feedback was provided for the entire duration of a run. Each session focused on only 1 technique parameter or speed, and the target zones were updated based on how well the participant could run within the target zones to ensure continuous challenges, but also to prevent demotivation due to too strict target zones. The wearable attempted to balance technique and intensity sessions to ensure that a maximum of 20% of the sessions were performed at higher intensities. Suggested changes were made relative to a baseline run, and only gradual changes of maximally 5% to 10% from this reference were made to ensure sufficient time for tissue adaptation. Note that the application did not provide a detailed training program, and runners could, therefore, self-determine when to run and how long they wanted to run.

### Outcome Measures

#### Primary Outcome

After each training session, each participant was required to complete a pop-up about any running-related pain/discomfort—using a numerical pain rating scale, ranging from 0 to 4, with 0 = no, 1 = light, 2 = mild to moderate, 3 = fairly severe, and 4 = severe pain/discomfort, respectively— and the location of the pain/discomfort—as indicated by clicking on a body part of a human body model. The locations were limited to major anatomic regions because self-reporting of tissue type and abnormalities (eg, differentiating between patellofemoral pain and patellar tendinopathy) is unreliable.^28^ If an injury was noticed before the start of a subsequent session and thus not recorded in the application, the participants were asked to report the injury to the researchers by email.

The primary outcome of interest was a running-related injury, which we a priori defined in 2 ways: (1) as a pain/discomfort rating of ≥1 for a similar body part for ≥7 consecutive days^69^ and (2) as any discomfort or pain, regardless of the number of days.^62^ We chose to compare the distance that was ran until the first injury between the groups, with the distance ran being recorded by the application. The injury rate was expressed per distance instead of time units as distance is a metric often used by runners in their training plan.

We performed 3 analyses to compare injury rates between the groups. First, the "intention-to-treat analysis” investigated whether the intervention (ie, real-time feedback) was more effective at reducing injury risk than no intervention when participants were analyzed according to the original/initial group allocation. The intention-to-treat analysis included any effect of adherence as participants who were originally allocated to the control group could have received real-time feedback if they did not turn off the feedback setting in the application, whereas participants allocated to the intervention group could have received no feedback if they turned off the feedback. To investigate the effect of the real-time feedback among participants who did actually receive real-time feedback, we performed 2 additional analyses: an “as-treated” and “per-protocol” analysis. The as-treated analysis investigated whether feedback effectively reduced injury risk regardless of whether participants were initially assigned to receive feedback.^58^ This analysis partially ignored the original randomization but had higher statistical power because of less-strict criteria. The per- protocol analysis investigated whether real-time feedback was more effective at reducing injury risk than no real-time feedback among participants who received the allocated treatment and were originally allocated to the intervention or control groups (ie, adhered to the assigned group).^58^ This analysis complied with the original randomization but has less statistical power than the as-treated analysis because of a smaller number of participants meeting both criteria. For both the per-protocol and the as-treated analyses, we a priori decided that the participants were included in the intervention group only when they ran ≥60% of their sessions with real-time feedback. We considered this threshold to provide a good balance between sufficient exposure to real-time feedback, while at the same time not being overly restrictive, which would limit the eventual sample size. We explored the sensitivity of our results to this decision by also analyzing thresholds of ≥50%, ≥70%, and ≥75% for allocation to the intervention group. In the as-treated analysis, we additionally allocated runners to the control group if they ran less than the specified percentage of sessions with real-time feedback. However, to minimize exposure in the control group to real-time feedback, these participants were allowed to run maximally 40% of their sessions with feedback, with an absolute maximum of 3 sessions with real-time feedback. This decision was based on research showing that coaching/feedback for >3 sessions can have long-term effects.^6^ Further details on this approach are provided in the Appendix (section 2.1; available online).

#### Secondary Outcomes

Running performance was measured as the self-reported personal best times of the runners in the year before the intervention up to a week after stopping the intervention. Motivation to exercise was assessed using the BREQ-2 questionnaire. Finally, we also explored the participants’ interest in continuing to use the wearable after the intervention study, as this can provide information on the long-term implementation of the wearable outside of a study setting. More details on the secondary outcomes can be found in the Appendix (section 2.2; available online).

### Data Analysis

#### Primary Outcome

Although the chi-square test is recommended to compare the proportion of injuries between groups in the CONSORT guidelines,^42^ it does not consider an individual's time at risk of sustaining an injury. It is, therefore, suboptimal for assessing the effects of the intervention on injuries.^16,47^ Therefore, a Cox proportional hazards regression was used to determine the difference in the rates at which injuries occurred between the groups. The distance between the baseline run and the date of the first injury or censoring was used to calculate the distance at risk, expressed in kilometers. A participant was right-censored if he or she dropped out due to an injury unrelated to running, disease, pregnancy, lack of motivation, or at the end of follow-up. The following covariates were a priori chosen to be included in the Cox regression model: group, age, sex, and body mass. As there were >50 injuries in both the intention-to-treat and the as-treated analyses, the inclusion of 4 covariates met the recommended 10 injuries per predictor variable included in the Cox regression to minimize bias of the regression weights.^47,51^ For the per-protocol analysis, there were 38 injuries regardless of duration and 18 injuries when considering only injuries >7 days in duration. We, therefore, included 1 and 2 predictors less in the models for these outcomes, respectively. Body mass was not included in the per-protocol analyses for all injuries regardless of duration, and body mass and sex were not included in the per-protocol analyses for >7-day injuries, as a backward regression showed these predictors to have the lowest contribution. Model assumptions were checked as specified in the Appendix (section 3.1; available online). Several guidelines recommend performing a 1-sided test when there is a directional hypothesis regarding the difference between groups.^29^ Our study had an a priori defined directional hypothesis that the group receiving real-time feedback would reduce injury rates and severity. Therefore, we used a 1-sided test to compare the injury rate between the groups. The directional hypothesis was motivated by the findings of studies showing that real-time feedback can be used to modify risk factors of running injuries^45,46^ and reduce actual injury risk (in a laboratory setting).^10^ For completeness, 2-sided *P* values and confidence intervals are also reported.

The first-time injury severity was considered a continuous variable and compared between groups using a linear model. Age, sex, and body mass were included as covariates in the model. Normality was assessed visually using quantile-quantile plots. A logistic regression analysis with age, sex, and body mass as covariates was used to determine the difference in the proportion of participants with multiple injuries between the groups. The injury location was descriptively reported for both groups.

#### Secondary Outcome

To compare the effects of the intervention on the motivation to exercise and running performance, we compared the change scores in different motivation subscales and the percentage change in self-reported personal best times (averaged over all distances within each participant) between the groups using a linear model, with age, sex, and body mass included as covariates. The correlation between weekly running volume and change in performance was investigated using a Pearson correlation coefficient. The interest of participants to continue using the wearable was reported descriptively.

## Results

**Figure 2.**
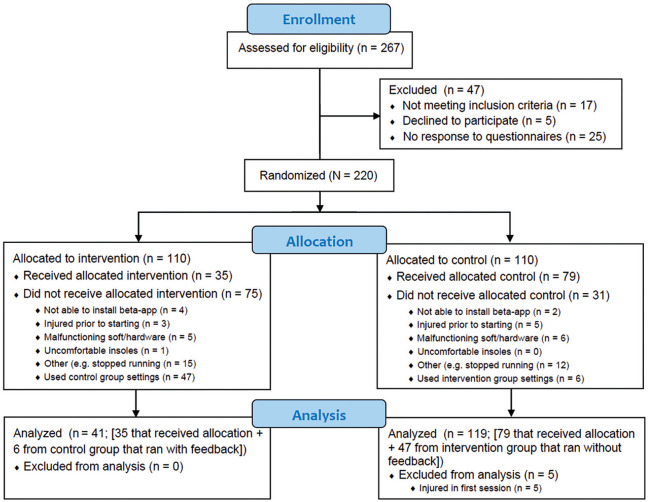
Participant enrollment, allocation, and analysis flowchart for the intention-to-treat, as-treated, and per-protocol analyses. For the intention-to-treat analysis, all participants were analyzed according to their original group allocation when they ran ≥1 session, resulting in a sample size of 172 runners (75 intervention, 97 control). Further, 35 (32%) runners originally allocated to the intervention group ran ≥60% of their training sessions with real-time feedback and were included in the intervention group in the as-treated analysis. On the other hand, 6 (5.5%) runners originally allocated to the control group ran ≥60% of their training sessions with real-time feedback and were also included in the intervention group, yielding a total sample of 41 participants in the intervention group. A total of 119 participants were included in the control group because 79 allocated to the control group ran with control sessions, while 47 runners allocated to the intervention group also ran with control group settings (ie, without feedback). In the per-protocol analysis, 35 runners were included in the intervention group and 79 in the control group.

A total of 267 runners were assessed for eligibility. An overview of the inclusion, allocation, and analysis processes is provided in Figure 2.

### Results of Intention-to-Treat Analysis

Baseline characteristics for all participants included in the intention-to-treat analysis are reported in Table 1. Table 2 details the baseline characteristics for runners who adhered to the assigned intervention group and those who were originally allocated to the intervention group but did not comply with this assignment. A comparison on the main demographic outcomes showed no significant differences between runners who complied and those who did not comply with the assigned intervention. The intention-to-treat analysis showed no significant differences in injury rates between the groups (Table 3).

**Table 1:** Baseline Characteristics for All Participants Included in the Intention-to-Treat Analysis^*a*^

	All	Intervention Group	Control Group
Characteristics			
No. of participants	172	75	97
Sex, M/F, %	61/39	55/45	66/34
Mean ± SD age, y	40.1 ± 10.8	39.2 ± 10.6	42 ± 10.9
Mean ± SD body mass, kg	71.6 ± 10.6	71.2 ± 10.6	76.9 ± 8.56
Mean ± SD height, cm	176 ± 8.70	175 ± 8.67	181 ± 7.15
Running			
Weekly training distance, km	8.22 (4.6-13.3)	8.12 (4.04-10.6)	8.26 (4.04-13.4)
Weekly participation in other sports, h	0.69 (0.07-1.59)	0.69 (0.22-1.31)	0.72 (0.07-1.59)

a Unless otherwise indicated, values are presented as mean ± SD when normally distributed or median (IQR) when nonnormally distributed. F, female; M, male.

**Table 2:** Characteristics of Participants in the Intervention Group Who Adhered to the Assigned Intervention and Participants Who Did Not^*a*^

	Adhered to Assigned Intervention	Did Not Adhere to Assigned Intervention	Mean Difference ± SE; *P*
Characteristics			
No. of participants	35	47	
Sex, M/F, %	52/48	55/45	
Mean ± SD age, y	38.8 ± 10.3	39.1 ± 10.9	0.26 ± 2.5; *P* = .92
Mean ± SD body mass, kg	70.5 ± 11.6	71.4 ± 9.88	0.84 ± 2.5; *P* = .74
Mean ± SD height, cm	175 ± 8.41	175 ± 9.13	0.22 ± 2.1; *P* = .92
Running			
Weekly training distance, km	11 (0.68-26.7)	8.31 (0.72-36.3)	−2.69 ± 1.7; *P* = .12
Weekly participation in other sports, h	0.71 (0.25-1.31)	0.69 (0.22-1.19)	−0.17 ± 0.17; *P* = .32

a Unless otherwise indicated, values are presented as mean ± SD when normally distributed or median (IQR) when nonnormally distributed. F, female; M, male.

**Table 3:** Cox Regression Results for the Primary Outcome According to the Intention-to-Treat Analysis^*a*^

Covariate	Model 1 (Unadjusted)		Model 2 (Adjusted)	
	Crude HR (95% CI)	*P*	Adjusted HR (95% CI)	*P*
All injuries^*b*^				
Group^*c*^	1.16 (0.68-1.99)	.59	1.11 (0.65-1.91)	.70
Sex^*d*^	Not included	-	0.70 (0.34-1.45)	.34
Mass, kg	Not included	-	0.99 (0.96-1.03)	.56
Age, y	Not included	-	0.98 (0.95-1.00)	.08
>7-day injuries^*e*^				
Group^*c*^	1.97 (0.91-4.11)	.09	1.90 (0.88-4.07)	.10
Sex^*d*^	Not included	-	1.68 (0.64-4.42)	.29
Mass, kg	Not included	-	1.02 (0.98-1.07)	.37
Age, y	Not included	-	0.97 (0.94-1.01)	.15

a Model 1 included only the group as a predictor, while model 2 included all predictors. HR values <1 indicate a lower injury (hazard) ratio. 95% CIs (lower–upper bound) and all *P* values are 2-sided. HR, hazard ratio.

b No. of injuries = 54; No. of censored participants = 104; total No. of participants in the analysis = 158 (2 censored before the earliest event). Total exposure = 26,999 km.

c Control group is reference.

d Male sex is reference.

e No. of injuries = 28; No. of censored participants = 129; total No. of participants in the analysis = 157 (2 censored before the earliest event). Total exposure = 32,415 km.

### Results of As-treated and Per-protocol Analyses

Baseline and follow-up characteristics, as well as reasons for dropout before study completion other than a running injury in the as-treated analysis, are reported in Tables 4 and 5, respectively. Injury locations are reported in the Appendix (available online).

**Table 4:** Baseline Characteristics for All Participants Included in the As-treated Analysis^*a*^

	All	Intervention Group	Control Group
Characteristics			
No. of participants	160	41	119
Sex, M/F, %	61/39	56/44	62/38
Mean ± SD age, y	40.2 ± 10.6	40.3 ± 10.9	40.1 ± 10.8
Mean ± SD body mass, kg	71.6 ± 10.7	72.1 ± 11.7	71.4 ± 10.2
Mean ± SD height, cm	176 ± 8.70	176 ± 8.40	177 ± 8.82
Running			
Weekly training distance, km	8.23 (4.59-13.7)	9.41 (6.47-15.6)	7.49 (4.14-13.1)
Weekly participation in other sports, h	1 (0-2)	1 (0-2)	1 (0-2)

a Unless otherwise indicated, values are presented as mean ± SD when normally distributed or median (IQR) when nonnormally distributed. F, female; M, male.

**Table 5:** Follow-up Characteristics for All Participants Included in the As-treated Analysis^*a*^

	All	Intervention Group	Control Group
Running-related characteristics			
Follow-up time, months	4.8 (1.56- 8.52)	4.9 (2.02-8.19)	4.7 (1.22-8.58)
Session duration, h	0.66 (0.52- 0.85)	0.66 (0.54-0.87)	0.69 (0.51- 0.84)
Session distance, km	6.99 (5.17-9.01)	7.31 (5.59-9.72)	6.98 (5.11- 8.61)
Weekly distance, km	8.23 (4.59- 13.7)	9.41 (6.47-15.6)	7.49 (4.14- 13.1)
No. of runs per week	1.23 (0.82- 1.88)	1.51 (0.93-2.03)	1.09 (0.81- 1.82)
Running speed, m·s^-1^	2.78 (2.59- 3.05)	2.78 (2.61-3.18)	2.78 (2.59-3)
Sessions within target zone, %	-	60.8 ± 17	-
Running surface, % of the time			
Concrete/asphalt	69.2 ± 26.7	65.2 ± 30.5	69.4 ± 25.9
Grass/sand/country roads	20.7 ± 21.9	19.5 ± 23.9	22.5 ± 22.2
Track	3.70 ± 8.10	4.12 ± 8.88	3.42 ± 8.20
Treadmill	0.48 ± 1.24	0.06 ± 0.24	0.76 ± 1.52
Injuries			
No. of injuries, % of total sample or group^*b*^	53 (33)	10 (24.4)	44 (37)
No. of injuries >7 days, % of total sample or group^*b*^	27 (16.9)	6 (14.6)	22 (18.5)
Injury incidence per 1000 running hours (95% CI)^*b*^	20.9 (15.9- 27.1)	12.6 (6.38-22.4)	24.1 (18.1- 32.8)
Injury incidence per 1000 running hours for >7-day injuries (95% CI)^*b*^	9.05 (5.87- 12.5)	7.33 (2.97-15.3)	9.23 (5.86- 13.9)
Participants with multiple injuries, % of total sample or group^*c*^	18 (11.3)	3 (7.32)	15 (12.6)
First-time injury severity, 0-4 Likert scale	1.34 ± 0.70	1 ± 0	1.42 ± 0.75
Reasons for dropout before 6 months	n = 92	n = 24	n = 68
Running-related injury	24 (26.1)	4 (16.7)	20 (29.4)
Non–running related injury^*d*^	2 (2.17)	2 (8.33)	00 (0)
Insoles were uncomfortable	7 (7.61)	1 (4.17)	6 (8.82)
App malfunctioning	43 (46.7)	11 (45.8)	32 (47.1)
Stopped running	00 (0)	00 (0)	00 (0)
Other/unknown, eg, COVID-19	16 (17.4)	6 (25)	10 (14.7)

a Unless otherwise indicated, values are presented as mean ± SD when normally distributed or median (IQR) when nonnormally distributed or n (%). COVID-19, coronavirus disease 2019.

b These numbers reflect first-time injuries only.

c Note that these present a combination of recurrent injuries (ie, injuries at the same location as the previous injury) and injuries at a new location.

d Examples include ankle sprain during soccer or back injury during house moving.

**Figure 3.**
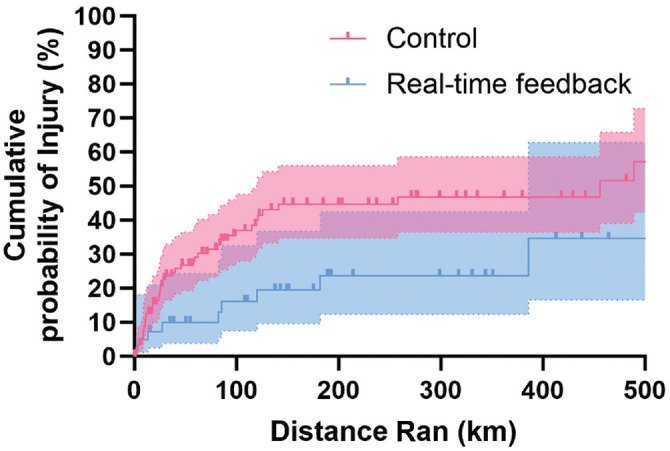
Nelson-Aalen curves show the cumulative probability of running-related injuries in the real-time feedback and control groups as a function of running distance in kilometers with the as-treated approach. Bold lines represent the estimated cumulative probability of an injury at each distance per group, while shaded areas represent the 95% CIs. The bold square symbols/thicks depict right-censoring (ie, dropout for nonrunning injury reasons).

When considering all injuries regardless of duration, the injury (hazard) rate in the as-treated analysis was significantly lower in the group that received real-time feedback when a 1-sided *P* value was used in line with the directional hypothesis (*P* = .03) (Figure 3). In contrast, when only injuries that lasted >7 days were included, there was no difference between the groups. Sensitivity analyses yielded similar effects for the other thresholds (Appendix Table A1, available online). The per- protocol analysis showed no significant differences in injury rates between the groups for all injuries regardless of duration and for injuries that lasted >7 days (Table 7).

The first-time injury severity was significantly lower in the group that received real-time feedback (–0.42; 1-sided *P =* .043) (Table 5). This difference remained significant after adjusting for covariates (–0.43; 1-sided *P =* .042). In contrast, the proportion of runners with multiple injuries did not significantly differ between groups for both the unadjusted (hazard ratio [HR], 0.83; 1-sided *P* = .40) (Table 5) and the adjusted model (HR, 0.82; 1-sided *P =* .40).

### Results of Secondary Analyses

Pre- and poststudy personal best times were provided by 20.5% (n = 43) of the sample. There was no significant improvement in self-reported personal best times for the intervention or control groups throughout the intervention period, and the change in personal best times from pre- to poststudy did not significantly differ between the groups in the model without additional covariates (3.65%; *P* = .15 (Table 8) or with additional covariates (3.07%; *P* = .26). Table 8 also shows the interest to continue wearable use after study completion and reasons for discontinued use. Other secondary results are described in the Appendix (available online).

## Discussion

The primary aim of this study was to investigate whether real-time feedback that aims to alter relative loading and relative speed by providing feedback on spatiotemporal metrics and speed using commercially available pressure-sensitive insoles is effective at reducing running injuries compared with no real-time feedback.

The intention-to-treat analysis showed no difference in injury risk between the intervention and control groups (see Table 3). This was expected because of the changes in group allocation for a substantial proportion of participants, nullifying any potential effects of the intervention. While changes in group allocation may provide information about the adherence and, thus, effectiveness of the wearable in-field, personal communication indicated that the change in group allocation primarily occurred because of confusion about the settings to use in the application. Specifically, all participants in the control group had to manually turn off the feedback feature before the start of the study. While we provided clear instructions on how to do this, some participants did not turn off the feedback feature and were, therefore, allocated to the intervention group. Similarly, some participants in the intervention group used the “quick-start” run button to start each session and, therefore, ended up in the control group as this option did not provide them with real-time feedback. Personal communication indicated that most of the participants did not deliberately use incorrect settings, and because the wearable normally provides feedback by default, the intention-to-treat analysis does not provide relevant information about the effectiveness of the wearable in-field. This is further supported by the absence of differences in major demographic characteristics between the runners who complied and those who did not comply with the intervention (see Table 2). Instead, it highlights the importance of ensuring easy-to-use applications to ensure compliance with wearables. Based on these findings, the as-treated and per-protocol arguably present a more informative analysis of the effectiveness of the wearable (Tables 6 and 7).

**Table 6:** Cox Regression Results for the Primary Outcome According to the As-treated Analysis^*a*^

Covariate	Model 1 (Unadjusted)		Model 2 (Adjusted)	
	Crude HR (95% CI)	*P*	Adjusted HR (95% CI)	*P*
All injuries^*d*^				
Group^*b*^	0.50 (0.25-0.99)	**.02** (.048)	0.53 (0.26-1.05)	**.03** (.07)
Sex^*c*^	Not included	-	0.77 (0.38-1.57)	.47
Mass, kg	Not included	-	0.99 (0.96-1.03)	.68
Age, years	Not included	-	0.98 (0.95-1)	.09
>7-day injuries^*e*^				
Group^*b*^	0.70 (0.28-1.72)	**.22** (.43)	0.70 (0.28-1.73)	**.22** (.44)
Sex^*c*^	Not included	-	1.70 (0.64-4.46)	.29
Mass, kg	Not included	-	1.02 (0.97-1.06)	.43
Age, years	Not included	-	0.97 (0.93-1.01)	.14

a Model 1 included only the group as a predictor, while Model 2 included all predictors. Also, 95% CIs (lower-upper bound) represent 2-sided CIs, and *P* values in parentheses represent 2-sided *P* values for consistency with the other covariates. Bold *P* values represent the 1-sided *P* value. HR values <1 indicate a lower injury (hazard) ratio. HR, hazard ratio.

b Control group is reference.

c Male sex is reference.

d No. of injuries = 54; No. of censored runners = 104; total No. of runners in the analysis = 158 (2 censored before the earliest event). Total exposure = 26,999 km.

e No. of injuries = 28; No. of censored runners = 129; total No. of runners in the analysis = 157 (2 censored before the earliest event). Total exposure = 32,415 km.

**Table 7:** Cox Regression Results for the Primary Outcome According to the Per-Protocol Analysis^*a*^

Covariate	Model 1 (Unadjusted)		Model 2 (Adjusted)	
	Crude HR (95% CI)	*P*	Adjusted HR (95% CI)	*P*
All injuries^*b*^				
Group^*c*^	0.66 (0.31-1.40)	**.14** (.28)	0.67 (0.32-1.43)	.**15** (.30)
Sex^*d*^	Not included	-	0.60 (0.30-1.22)	.16
Mass, kg	Not included	-	Not included	-
Age, y	Not included	-	0.97 (0.94-1.01)	.10
>7-day injuries^*e*^				
Group^*c*^	1.33 (0.50-3.59)	N/A (.57)	1.29 (0.48-3.50)	N/A (.62)
Sex^*d*^	Not included	-	Not included	-
Mass, kg	Not included	-	Not included	-
Age, y	Not included	-	0.97 (0.92-1.02)	.17

a Model 1 included only the group as a predictor, while Model 2 included all predictors. Also, 95% CIs represent 2-sided CIs (lower-upper bound), and *P* values represent 2-sided *P* values for consistency with the other covariates. Bold *P* values represent the 1-sided *P* value. For >7-day injuries, we did not apply a 1-sided *P* value, as the direction of the effect was opposite of that hypothesized. HR values <1 indicate a lower injury (hazard) ratio. HR, hazard ratio; N/A, not applicable.

b No. of injuries = 38; No. of censored runners = 72; total No. of runners in the analysis = 110 (1 censored before the earliest event). Total exposure = 20,043 km.

c Control group is reference.

d Male sex is reference.

e No. of injuries = 18; No. of censored runners = 91; total No. of runners in the analysis = 109 (1 censored before the earliest event). Total exposure = 24,036 km.

In partial support of our primary hypothesis, the as-treated analysis showed a significantly lower injury rate and severity in the group that received real-time feedback than the group that did not receive real-time feedback when any injury or discomfort, regardless of the duration, was used as outcome. The effect magnitude also increased with more sessions run with feedback relative to total sessions, further strengthening the hypothesis that feedback might have explained this effect (Appendix Table A2, available separately). There were no significant differences between the groups when comparing injuries that lasted >7 days or in the proportion of runners with multiple injuries. However, both showed nonsignificantly lower injury rates and a nonsignificantly smaller proportion in runners with multiple injuries in the intervention group. Although the per-protocol analysis showed no significant difference in injury rates (likely because of a smaller sample size), the direction of the effect was similar when comparing injuries regardless of their duration (Table 7). Self-reported personal best times and motivation to exercise did not significantly change within each group over the course of the study, and changes did not differ between the intervention and control groups.

The significant reduction in injury rate with real-time feedback found in the as-treated approach agrees with a previous laboratory-based study where the injury rate was significantly lower during the 1-year follow-up in the group that received feedback to reduce the vertical impact peak by softening their footstrike.^10^ However, the lower injury rate partially contrasts with a previous in-field study that found no overall effect of wearable-based real-time feedback that aimed to modify a rearfoot strike to a nonrearfoot strike on the incidence rate of running injuries.^44^ There are several possible reasons for this discrepancy. First, the researchers attempted to modify footstrike from a rearfoot strike to a nonrearfoot strike, and it is debatable whether this is beneficial to reduce overall injury rates.^3^ Indeed, while such a strategy may decrease the risk of knee injuries, it can also increase the risk of foot and Achilles tendon injuries,^10,44^ leading to no net reduction in injury rate. In contrast, the wearable used in the present study aims to gradually and incrementally modify spatiotemporal metrics relative to a runner's self-selected gait; for example, by modifying a runner with a very pronounced rearfoot strike toward a less pronounced rearfoot strike, while a runner with a very pronounced forefoot strike is gradually modified toward a less pronounced forefoot strike. Such a strategy may reduce the load on specific body segments; for example, the knee or foot/Achilles, respectively, while the loads on other structures are not substantially increased, potentially reducing overall injury risk. In indirect support of this, while other studies have observed a shift toward higher Achilles/foot injuries with gait retraining methods that aimed to increase cadence or change an individual's gait to adopt a forefoot strike,^10,44^ we did not observe a clear or pronounced increase in the injuries at these locations (Appendix Table A1). However, we did not have sufficient statistical power to test this. Further research is required to substantiate whether this can be attributed to an overall more optimally distributed load because of real-time feedback. Second, the self-reported compliance in the intervention group in a study by Morris et al^44^ was only 14%, which could also have contributed to the ineffectiveness of the intervention. The low compliance could be due to the participants’ inability to freely choose their running pace, having to wear earphones to receive real-time feedback, and having to attach an accelerometer to the tibia during each run. In contrast, runners in the present study could freely choose their training pace, did not need to wear earphones, and did not need to mount an accelerometer to the shoe. However, they were required to use instrumented insoles and bring their smartphone during each run. While ~8% (see Table 5) of the participants dropped out during the study because the (prototype) insoles were considered uncomfortable (typically the cable connecting the insoles with the pod), the ability to freely choose the running pace and easier use of insoles as compared with a tibial mounted accelerometer may have increased the overall compliance with the intervention. Third, Morris et al did not provide feedback on the relative intensity of the training sessions, which could have led to some participants’ training too hard.^2,64^ This may, in turn, lead to exacerbated fatigue^7^ and further contribute to the ineffectiveness of the intervention. In contrast, the application in our study incorporated periodic “stable speed run” sessions (Appendix Table A5) that aimed to maintain the running speed within 5% of the comfortable running speed as determined with the talk test during the baseline run.

No significant differences were observed between the groups when considering only injuries that lasted >7 days in both the as-treated (Table 6) and the per-protocol (Table 7) analyses. This is in line with the previously discussed studies, as Morris et al^44^ only included injuries that lasted >7 days and found no significant effect of wearable-based feedback on the incidence rate, while Chan et al^10^ defined an injury as the absence of training for >2 days and did find a significant reduction in injury rates—in line with our study when including all injuries regardless of their duration. Nevertheless, a reduction in injuries with a duration of ≤7 days may still be considered relevant from a health, performance, and psychological perspective. For example, discomforts or pain that do not require longer-term stoppage may still lead to reductions in training intensity or volume by forcing runners to complete shorter or fewer runs, decreasing the health benefits of running and running performance.^23,32^ Furthermore, such shorter-duration injuries may also lead to psychological distress.^39,41^ Moreover, the severity of the first injury (ie, of all injuries regardless of duration) was significantly lower in the group that received real-time feedback (–0.43; 1-sided *P* = .042). This may have resulted from a reduced load and damage due to biomechanical gait modifications in response to the feedback, although further research is required to substantiate this notion. Combined with the lower injury rates observed for all injuries regardless of duration, these findings suggest some utility of the wearable to reduce overall injury burden and associated dropout.

A second aim of our study was to assess whether the feedback provided by the wearable led to larger increases in performance and motivation. Specifically, some studies have shown that a shorter contact time is associated with better running economy,^56,61^ and the wearable, therefore, aims to improve performance; for example, by indirectly reducing ground contact time relative to flight time via real-time feedback on cadence and footstrike. However, self-reported running performance as assessed by personal best times did not improve in either group, resulting in no significant difference between groups in the change in performance from pre- to poststudy (Table 8). This suggests that any potential alterations in running technique that may have contributed to a lower injury risk did not result in changes in performance. This may be because the overall body of evidence shows that the contact time or the duty factor (contact time to flight time ratio) are not associated with running economy,^65^ and potential alterations in (relative) contact time may, therefore, not have altered running economy. Nevertheless, any potential alterations in running technique did not negatively affect performance, as observed in some studies that showed gait retraining impaired running economy.^14,21^ However, as performance is determined by multiple (physiological) variables in addition to running economy, further experiments in controlled conditions are required to investigate the effectiveness of the real-time feedback on running economy.

**Table 8:** Secondary Outcomes^*a*^

Outcome	All	Intervention	Control
Performance	n = 43	n = 17	n = 26
Change in personal best times,^**b**^ %	−2.13 ± 8.13	0.07 ± 6.34	−3.57 ± 8.94
Continuation of wearable use after study completion	n = 53	n = 19	n = 34
Participants who intended to continue using the wearable after study completion, %	58.5	61.8	52.6
Reasons for discontinued use after study completion, %	n = 22	n = 13	n = 9
I don't understand the feedback^*c*^	22.7	0	38.5
I don’t think the feedback is useful	13.6	33.3	0
I don’t think the measurements are accurate	0	0	0
I got bored with the application/feedback	9.09	11.1	7.69
I don’t find the equipment comfortable	13.6	0	23.1
I don’t want to put the pods/insole on/in the shoe each time I go for a run and for charging	4.55	0	7.69
The equipment breaks too quickly	18.2	22.2	15.4
I don’t like to run with a phone	18.2	33.3	7.69

a Data are presented as mean ± SD or %.

b Negative changes indicate performance improvements (ie, shorter times to complete a given distance).

c Note that this referred to the postsession feedback for the control group or a combination of real-time and postsession feedback for the intervention group. For the control group, only a summary of the recorded metrics was shown (eg, mean contact time) without details on how to interpret the values.

A low motivation to run has been suggested to also contribute to dropout.^64^ A second way by which wearables may contribute to lower dropout rates is by improving motivation to exercise; for example, by increasing perceived competence using real-time feedback or simply by tracking running distance and speed so that runners can use this information for goal setting.^12^ However, motivation to exercise did not significantly change for either the intervention or the control groups from pre- to poststudy, and the change did not differ between the groups (Appendix Table A3, available online). The absence of change in motivational outcomes may be because the baseline Behavioral Regulation in Exercise Questionnaire–2 (BREQ-2) scores on identified and intrinsic motivation were already relatively high, while scores on extrinsic motivation–related scales were low compared with nonactive runners,^54^ allowing only a small potential for change. Nevertheless, this suggests that the wearable has no effect on the motivation to run in recreational runners, and it may rather prevent dropout by reducing injury burden. Importantly, injuries were also the primary reason for dropout in our study, after wearable malfunctioning problems, while low motivation scores, as assessed by the BREQ-2 questionnaire, showed no significant difference between runners who dropped out for noninjury reasons and those who did not drop out (Appendix Table A4, available online). This is in line with previous findings among novice runners.^26^ Approaches to reduce dropout in both novice and recreational runners may therefore primarily need to focus on reducing injuries.

Previous studies have shown that exercise interventions that effectively reduce injury rates in a well-controlled study setting may not be implemented in practice, thus reducing their effectiveness.^63^ Based on these findings, we investigated whether the participants who completed the study were interested in continuing to use the wearable after study completion. Overall, among those who completed the study and filled in the final questionnaire, ~60% indicated they were interested in continuing to use the (prototype) wearable after the study (Table 8). We explored the reasons for discontinued use because a better understanding of this may assist in ensuring more successful uptake in the future.^35^ Reasons for discontinuation differed between the groups, with the primary reason for discontinuation in the control group being that they did not understand the feedback provided in the application upon session completion. As the feedback upon session completion for the control group provided only an overview of the recorded biomechanical metrics without advice on how to use them, this illustrates the importance of feedback that specifies how to use the recorded metrics to optimize performance/health or minimize injury risk to ensure adoption in practice.^64^ In support of this, none of the runners in the group that received real-time feedback with specific instructions on what to modify reported an inability to understand the feedback as a reason for discontinued use. Within the intervention group, the most common reasons for discontinued use were that the feedback was not considered useful (33%) and that participants did not like to run with a phone (33%). The participants who did not consider the biomechanical feedback useful indicated that they would have preferred “feedback” concerning a training plan instead of running technique–related feedback. This finding is consistent with previous studies, showing that recreational runners typically use simple metrics such as distance and speed for motivational purposes. In contrast, more advanced runners also prefer more advanced biomechanical metrics.^12^ Wearables aimed at recreational runners may, therefore, implement training plans and biomechanical feedback to improve wearable uptake in practice. Furthermore, providing feedback via other devices (eg, a smartwatch) as opposed to a phone would also ensure further uptake, in line with previous research.^12,34^ Equipment durability issues also comprised an important reason for dropout in the intervention group (22%) (Table 8). When these findings are combined with the observation that the primary reason for dropout before 6 months (47% of the total sample) were issues with the prototype wearable application (eg, crashing, loss of Bluetooth connection, or other software bugs) (see Table 5), these findings primarily highlight that further improvements to the wearable hardware and software might have the largest effect on ensuring implementation in practice. In this regard, it should also be emphasized that most participants ran with a prototype wearable and beta application, which may have contributed to the hardware and software issues. Notably, while inaccurate metrics are an important reason for discontinued use of wearables,^36,60,64^ none of the runners in the present study reported perceived inaccuracy of the metrics as a reason for dropout (Table 8). This is in line with the high accuracy and reliability of the wearable in laboratory conditions for measuring spatiotemporal outcomes.^66^

### Methodological Considerations

Several methodological considerations should be considered when interpreting the findings of this study. Strengths of this study include the relatively large sample size, prospective follow-up over a minimum of ~6 months, the in-field setting, the use of a wearable that did not restrict running speed, and a sensitivity analysis on the primary outcome. A first consideration relates to the use of self-reporting of injury status. Ideally, all injured runners should have attended a clinical examination to validate the injury diagnoses. However, this would have reduced the feasibility of the study. To improve the validity of self-reporting, the app showed a visualization of the body, where the participants could select where they felt pain/discomfort. Injury data were limited to the approximate location of pain, as self-reporting of tissue type and abnormalities (eg, differentiating between patellofemoral pain and patellar tendinopathy) has been shown to be unreliable.^28^ A second consideration relates to the determination of training intensity using the talk test. While the talk test setup we used has several limitations—such as errors because of head wind and no control over whether participants regularly reperformed the talk test to ensure an appropriate training prescription^27^—we purposely choose the talk test over other methods to prescribe training intensity (eg, percentage of estimated maximum heart rate or 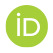), as the error with these methods might be even larger on an individual level.^18,38,48,57^ A third consideration is related to issues or crashing of the wearable application for several participants. This could have led to an underestimation of the distance run in some instances, and thus the distance and time at risk. Yet, we expect this underestimation to be similar in both groups and to mostly affect the overall injury incidence. Nevertheless, the observed overall incidence of 9.05 injuries per 1000 hours for injuries >7 days is slightly higher than the incidence among recreational runners reported by a systematic review (7.7 injuries per 1000 hours) in recreational runners,^67^ suggesting any underestimation to be small. A fourth consideration relates to the wearable. The strengths of the wearable include its relative ease of use compared with more cumbersome wearables, such as tibial mounted accelerometers and its ability to provide real-time feedback via an application on a mobile phone. There are, however, some limitations to the algorithms implemented within the wearable. Specifically, although methods have been developed to estimate absolute tissue loading from running wearables using various statistical methods,^4,8,22^ the wearable used in this study did not measure the *absolute* load on tissues but rather inferred a *relative* loading distribution from correlations with spatiotemporal metrics reported in the literature. Such an approach may introduce errors at the individual level (eg, because of the differences in tissue properties or geometry), which in turn may reduce the effectiveness of feedback. Further, the version of the wearable used for this study was limited to providing feedback on cadence, footstrike, and relative speed. While cadence manipulations may be beneficial for improving running economy^65^ and reducing loading/damage on common injury locations in recreational runners,^20^ and while footstrike manipulations have also been shown to be effective at reducing the cumulative patellofemoral impulse^17^ and Achilles tendon peak strain,^37^ feedback on other factors (eg, vertical oscillation because of its association with running economy^65^) may also be of benefit to runners. However, some of these other factors may be less unintuitive to manipulate for runners.

A final but important consideration relates to the change in group allocation for some participants because of the use of different settings in the application. To ensure we could include many participants, we performed an as-treated Cox regression whereby participants were analyzed according to the eventual group they ended up in. This meant that 33% of the participants included in this analysis were not originally allocated to the specific group. Although the overall group characteristics were still largely similar (see Table 4) and further controlled for by including important characteristics as covariates in the regression analysis, there may be other (unmeasured) confounding factors that could bias this analysis. For example, there may be an undefined characteristic specific to the participants who changed group allocation that influenced their injury risk. To explore how the change may have influenced group allocation, we also performed a per-protocol Cox regression whereby only participants who ran in the group to which they were originally randomized were included (see Table 7). This analysis had substantially fewer participants and thus lower statistical power. In addition, it included fewer covariates because of the smaller number of events. When combined with the potentially selective loss of participants, this analysis may be biased.^58^ Nevertheless, the analysis revealed a similar mean injury rate ratio when considering any injury regardless of duration, thus providing further confidence in the results obtained with our as-treated analysis. However, for injuries with a duration of >7 days, the direction of the effect obtained with the per-protocol analysis was reversed compared with the as-treated analysis (Tables 6 and 7). Because of the very small number of events and smaller number of covariates included in this latter analysis, this finding should be interpreted with great caution.

### Implications

Numerous studies have attempted to reduce running injuries, for example, using a graded running program^9,53^ or an online information program,^13,25^ but most approaches so far have been ineffective. In contrast, gait retraining methods have shown promising effects in a laboratory-based setting^5,10^ but have so far not been effective when applied in-field.^44^ By combining real-time feedback on both spatiotemporal metrics and relative speed, the wearable used in the present study reduced injury rates and injury severity. Reducing overall injury burden can enable runners to continue a physically active lifestyle.^15,55^ This is of great importance from a public health perspective because physical activity—particularly running—reduces the risk of numerous psychological and physical health conditions.^50^ An important consideration regarding the implementation of the wearable to reduce injury risk is that a significant number of participants (47%) dropped out from the study because of issues with the wearable application, suggesting that further software improvements are required to ensure adoption in practice. It is important to note that these issues can occur in prototypes and may be reduced in a commercial version.

## Conclusion

This study shows that real-time feedback on spatiotemporal metrics and relative speed provided by commercially available instrumented insoles may reduce the rate and severity of injuries in recreational runners, leading to less dropout. The feedback did not influence running performance and motivation to exercise.

## Supplemental Material

sj-pdf-1-ajs-10.1177_03635465231222464 – Supplemental material for The Effect of Wearable-Based Real-Time Feedback on Running Injuries and Running Performance: A Randomized Controlled Trial Supplemental material, sj-pdf-1-ajs-10.1177_03635465231222464 for The Effect of Wearable-Based Real-Time Feedback on Running Injuries and Running Performance: A Randomized Controlled Trial by Bas Van Hooren, Guy Plasqui and Kenneth Meijer in The American Journal of Sports Medicine
