# Supplementary material for: The Effect of Wearable-Based Real-Time Feedback on Running Injuries and Running Performance: A Randomized Controlled Trial
Source: Am J Sports Med. 2024 Jan 29;52(3):750–65. doi: 10.1177/03635465231222464 (PMC10905988; doi:10.1177/03635465231222464)
Supplement: sj-pdf-1-ajs-10.1177_03635465231222464 – Supplemental material for The Effect of Wearable-Based Real-Time Feedback on Running Injuries and Running Performance: A Randomized Controlled Trial [file sj-pdf-1-ajs-10.1177_03635465231222464.pdf]

The effect of real-time relative load and speed feedback by pressure-sensitive insoles on running injuries and running performance: A randomized controlled trial

Appendix

1. Methodological details

1.1 Sign-up process

During the recruitment period, a total of 437 individuals signed up via a website created for this study. This website contained general information about the study procedures. From the 437 signed-up individuals, the first individuals to sign-up were sent a questionnaire to check the in- and exclusion criteria until the desired sample size was met, and were asked to sign an informed consent form before completing a baseline questionnaire. This questionnaire collected information on age, height, body mass, sex, past injuries, current weekly running distance and frequency, personal best times, and running shoes.

1.3. Equipment

Figure A1 depicts the commercial and prototype version of the insoles that participants received. The prototype version differed from the commercial version by the use of 3D printed shells that were manually glued to create the pod housing as opposed to an ultrasonically welded injection molded ABS plastic pod in the commercial version. Additionally, the commercial version used a slightly different design of the insoles, cable and connectors. The thickness and flexibility of the cables was however similar. Note that the difference in size of the insoles is merely used to illustrate the different sizes that were provided. In the image, sizes M (medium) and XL (extra-large) are depicted next to each other, with 4 sizes available in total (S, M, L, XL), that will fit into EU size 36-48. The application installed on the phone was also a prototype beta-application for most participants. This application differed from the commercial application in lay-out and functionality. While the sampling frequency of the pressure sensors could be configured up to 250 Hz in the app, a sampling frequency of 150 Hz was used as this has been shown to provide sufficient accuracy[1], while also prolonging battery life.

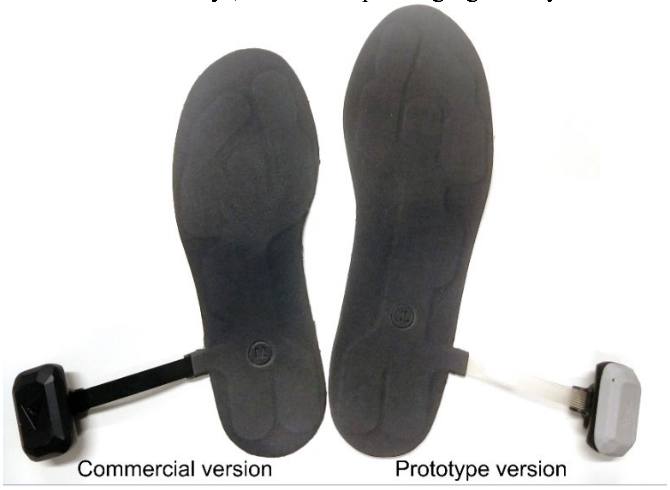

Figure A1. Commercial and prototype version of the pressure-sensitive insoles (of different size).

1.4. Participant instructions

Prior to the 6-month intervention, all participants were provided with documents and shown videos with information on how to place the instrumented insoles in the shoes, attach the inertial measurement unit to the shoes, and how to use the mobile phone application. The videos were available from the company website. All intervention and control participants were informed to strictly adhere to the training guidelines (see below) and to not share their app and insoles with other users to prevent bias in the collected data. Participants wo shared their app and insoles were excluded from all analyses. Further, prior to the intervention the participants in both groups were requested to avoid using other wearables that provided real-time feedback on their running technique or intensity of exercise and the adherence to this request was monitored using a questionnaire at the end of the study.

1.5. General training guidelines

All participants were provided with the following general training guidelines prior to the start of the study: 1) run at least twice a week; 2) plan at least one day of rest between the training sessions when possible; 3) you are allowed to increase the number of training sessions per week, but try to limit this to maximally one extra session per two months compared to your current training frequency; 4) you may gradually increase your weekly distance over the duration of the study, but increase your weekly distance by maximally 10% of your current weekly distance, and 5) run at least 3 km per session. Participants in the intervention group were also instructed to run most sessions at a comfortable speed as determined by a talk-test.

To ensure an optimal training stimulus, and to improve compliance with the running program, each participant performed the training program at their own relative intensity, but only the participants in the intervention group received real-time feedback on the relative intensity during some sessions. The relative intensity was based on the highest running speed during which participants could still comfortably talk as assessed using a talk-test, performed during the baseline run. In this run, the participants were required to run while counting out loud from 0-30 and rate if talking was 1) comfortably, 2) not fully comfortably/equivocal, 3) not comfortably.[2] If talking was rated as not (fully) comfortable, they were instructed to reduce the running speed to a speed at which they could talk comfortably, continue running at the reduced speed for 3 minutes and repeat the procedure in the last 30 seconds.[2] If talking was comfortable, they were instructed to increase the speed until the talking was rated as equivocal. The average speed during the run that included the talk test was recorded by the app. In training sessions that focussed on running at a comfortable speed, the speed from the baseline run with a range of ± 5% was used as the target training speed for feedback, while a range of up to 20% was used during interval sessions. Participants were instructed to perform the baseline run on a flat concrete/asphalt or equivalent road of at least 1 km and pick a day without strong head or tail wind and a temperature between 5 and 20 degrees Celsius. Further, they were asked to re-perform the baseline run with talk-test every four weeks to ensure a continuous optimal training intensity, or to manually re-perform the talk-test at any time to adjust the raining intensity if required.

2. Outcomes

2.1. Primary outcomes

Figure A2 provides an overview of the number of sessions ran with real-time feedback (green) and no real-time feedback (grey) for each participant. In this figure, individuals were only included in the intervention group when they ran ≥60% of their sessions with real-time feedback.

For some individuals, this meant that some sessions performed without real-time feedback at the end of the intervention period were not included in the distance at risk (and thus not shown in the figure). Control sessions were only included in the distance at risk up to the point where maximally 40% of the sessions were performed with real-time feedback, or when an absolute maximum of three sessions were performed with real-time feedback. An example of this procedure is provided in Figure A3.

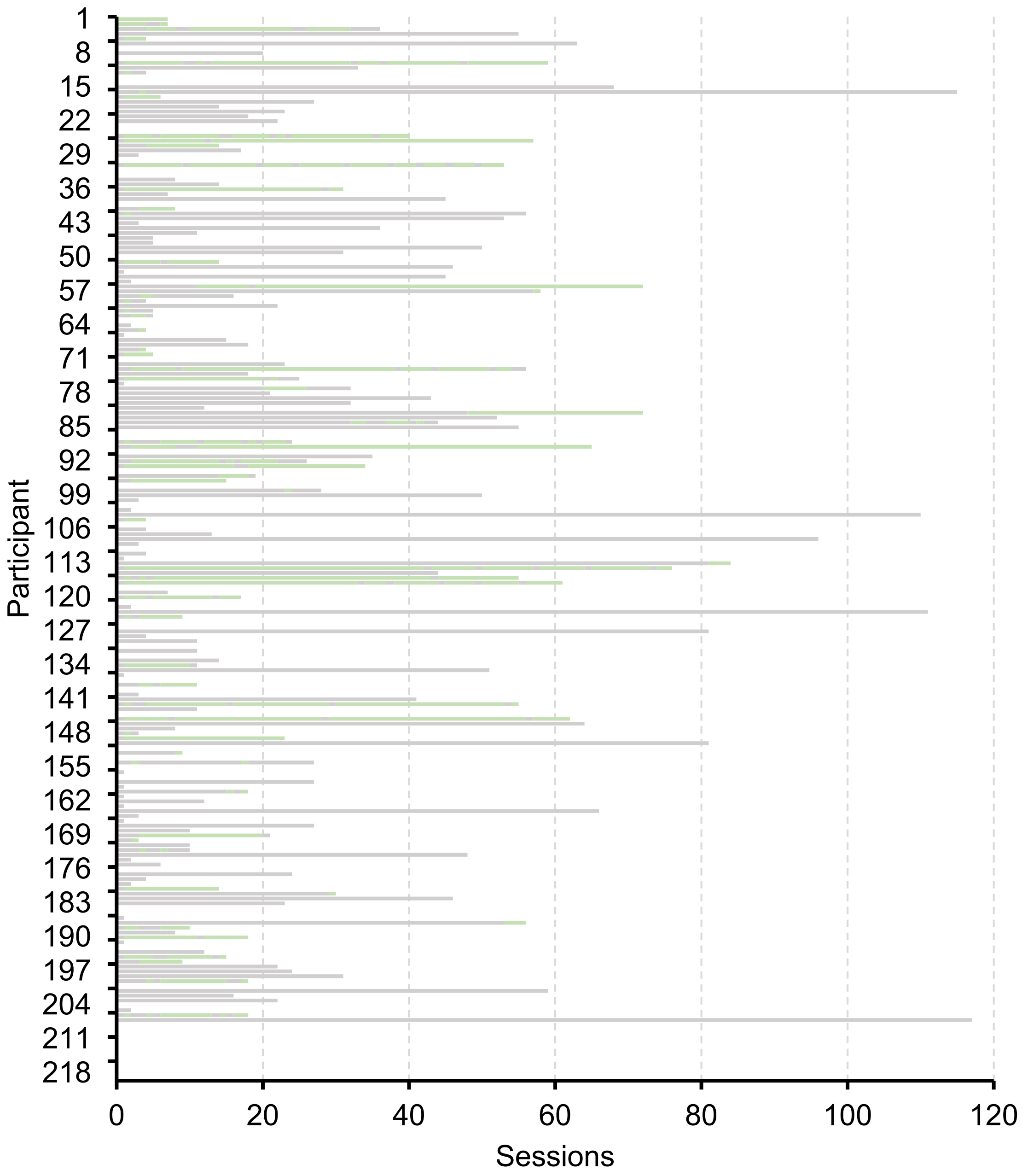

Figure A2. Overview of the number of sessions ran with real-time feedback (green) and no real-time feedback (grey) for each participant. In this example, individuals were only included in the intervention group when they ran >60% of their sessions with real-time feedback. Control sessions were only included in the distance at risk when maximally three sessions were performed with real-time feedback and when the number of intervention sessions was maximally 40% of the total sessions.

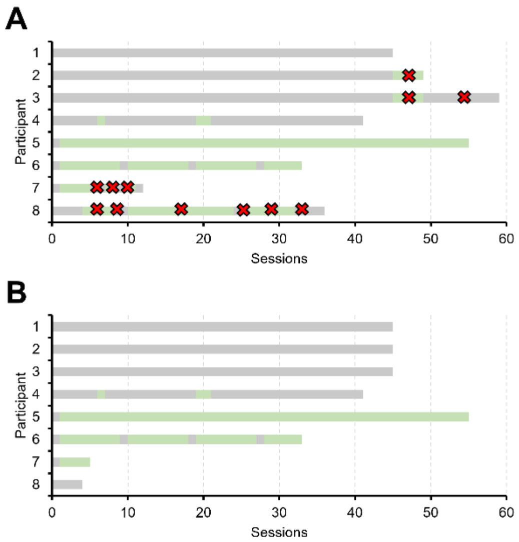

Nr	Control	Feedback	Control	Feedback	Control	Feedback	Control	Feedback	Final group
1	45								Control
2	45	4							n.a.
3	45	4	10						n.a.
4	6	1	12	2	20				Control
5	1	54							Intervention
6	1	8	1	8	1	8	1	5	Intervention
7	1	4	2	1	2	1	1		n.a.
8	4	4	2	14	2	6	4		n.a.

Nr	Control	Feedback	Control	Feedback	Control	Feedback	Control	Feedback	Final group
1	45								Control
2	45								n.a.
3	45								n.a.
4	6	1	12	2	20				Control
5	1	54							Intervention
6	1	8	1	8	1	8	1	5	Intervention
7	1	4							n.a.
8	4								n.a.

Figure A3. Example of sessions included in the distance at risk for statistical analysis. A shows the original (hypothetical) dataset with each line depicting a participant and colors depicting whether the participant ran with (green) or without (grey) real-time feedback during a particular session. B shows the modified dataset after deleting sessions counting towards the distance at risk to meet the inclusion thresholds for the intervention and control groups. The text below provides a more detailed rationale for each example participant.

Participant 1 has ran 45 sessions without feedback (100%) and is therefore included in the control group in all analysis.

Participant 2 has ran 45 sessions without feedback (92%), but also 4 sessions with feedback at the end of the observation period. While the threshold of percentage of control to intervention sessions is met (i.e., ≥60%; or vice versa <40% intervention sessions), four sessions with feedback is higher than the allowed three sessions to be included in the control group. By deleting the four sessions at the end of the observation period from the time at risk, this participant can be included in the control group in all analysis.

Participant 3 has ran 45 sessions without feedback, then four sessions with feedback and another 10 sessions without feedback. While the threshold of percentage control to intervention sessions is again met (i.e., ≥60%), four sessions with feedback is higher than the allowed three sessions to be included in the control group. By deleting the four sessions at the end of the observation period and subsequent 10 control sessions (as these may be influenced by the four sessions ran with feedback) from the time at risk, this participant can be included in the control group in all analysis. If the participant would have experienced an injury during any of the sessions with feedback, or subsequent sessions without feedback, this injury would therefore also not be included in the analyses and the participant would thus be right-censored prior to running with the intervention settings.

Participant 4 has ran a total of 41 sessions, of which three with feedback. Since the threshold of percentage control to intervention sessions is again met (i.e., ≥60%), and because only three sessions were performed with feedback, this participant can be included in the control group.

Participant 5 has ran a total of 55 sessions, and only one session was without feedback. This yields a percentage of ~98% of intervention sessions of total sessions and this participant can therefore be included in the intervention group for all analysis (i.e. for all thresholds used in sensitivity analysis).

Participant 6 has ran a total of 33 sessions, and only four sessions were without feedback. This yields a percentage of ~88% of intervention sessions of total sessions and this participant can therefore be included in the intervention group for all analysis (i.e. for all thresholds).

Participant 7 has ran a total of 12 sessions, but 6 sessions were without feedback. This yields a percentage of 50% of intervention sessions of total sessions and this participant can therefore only be included in the intervention group for the thresholds were ≥50% of the sessions needs to be performed with real-time feedback. For the other thresholds, the last sessions will be deleted from the distance at risk until ≥60%, ≥70%, or ≥75% of the remaining sessions are performed with real-time feedback.

Participant 8 has ran ≥60% of the session with feedback and can therefore be included in the intervention group with this threshold and for the ≥50% threshold. However, the ≥70% threshold is not met. By deleting sessions, starting at the last sessions, it is not possible to achieve ≥70% intervention to control ratio, thus in the end requiring allocation to control group for analysis with this threshold.

The thresholds whereby ≥50%, ≥60%, ≥70%, and ≥75% of the sessions had to be performed with real-time feedback in order to be allocated to the intervention group meant that a minimum number of two (to yield ≥50% sessions with feedback with one baseline session), three (to yield ≥60% sessions with feedback with one baseline session), or four sessions (to yield ≥75% sessions with feedback with one baseline session), would need to be performed to be allocated to the intervention group. This therefore meant that individuals that got injured during for example the first session could not contribute towards the injury risk in the intervention group, and would instead count towards the injury risk in the control group.

This approach is prone to survivorship bias, whereby only individuals that remained uninjured in the first few sessions could be allocated to the intervention group. This in turn causes the intervention group to show fewer injuries than the control group. To minimize survivorship bias, we therefore did not include any individuals that got injured during the first session (in case of a ≥50% threshold for intervention group allocation), first two sessions (in case of a ≥60% threshold) or three sessions (in case of a ≥70% threshold). This procedure is clarified in Figure A4.

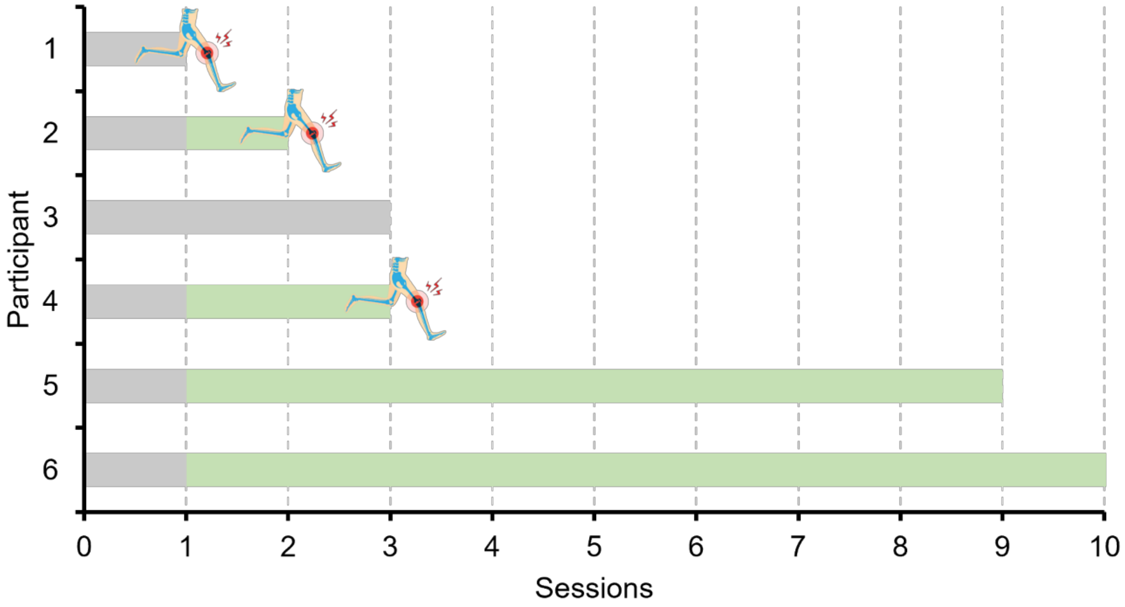

Figure A4. Example of injuries included and excluded in the distance at risk for statistical analysis. For the ≥50% threshold analyses, ≥50% of the sessions should be performed with real-time feedback to be allocated to the intervention group. Because all participants first performed a baseline session without feedback, this meant that at least two sessions had to be performed to be allocated to the intervention group with this threshold (e.g. as in participant 2). As a consequence, any injuries that occurred during the baseline run (participant 1) would always increase injury rate in the control group, thus creating more favorable outcomes for the intervention group by survivorship bias. By excluding any injuries that occurred in the baseline run, we removed this potential bias by applying equal criteria to both groups. Similarly, when using the ≥70% threshold, at least three out of four sessions should be performed with real-time feedback to be allocated to the intervention group. The injury that occurred in participant four could therefore not contribute to the injury rate in the intervention group because the minimum threshold of ≥70% of the sessions ran with feedback was not met (as two out of three sessions yields a percentage of 66%). However, because the participant did also not perform ≥60% of the sessions without feedback, this injury could also not be included in the control group. In this case, the participant was therefore excluded from analyses.

2.2. Secondary outcomes

Running performance was considered an important secondary outcome since a lack of improvement in running performance may result in feelings of incompetence and hence decreases of intrinsic motivation. Running performance was therefore measured as the self-reported personal best times of the runners in the year prior to intervention up to a week after stopping with the intervention.

A lack of motivation has been suggested to be an important secondary reason for dropout in running in addition to injuries.[3] We therefore also investigated if the real-time feedback lead to larger increases in motivation to exercise than the control group. Motivation was determined before and after the intervention using an online questionnaire (Behavioral Regulation in Exercise Questionnaire-2 [BREQ-2].[4] We also explored if individuals that dropped-out for other reasons than an injury (both running and non-running related) exhibited lower motivation in the pre-study questionnaire than non-dropouts.

Finally, we also explored the interest of participants to continue running with the ARION equipment after the intervention study as this can provide information on the long-term implementation of the wearable outside of a study setting.

3. Data analysis

3.1. Primary outcomes

Confounding was investigated using multicollinearity diagnostics (variance inflation factor <10), while the assumption of proportional hazards (i.e., constant ratio of hazards) was evaluated by log-minus-log plots. The difference in injury (hazard) ratios between groups was assessed using a one-sided *p*-value as we *a priori* hypothesized the intervention group to show lower injury rates compared to the control group. The linearity assumption was assessed by plotting the continuous covariates against Martingale residuals of the null Cox model.

4. Additional results and sensitivity analyses

4.1. Injury locations

Table A1 shows the injury locations for all injuries regardless of their duration across participants combined, and separately for each group.

Table A1 Injury locations for all participants combined, and separately for each group

Location	All (% of group total)	Intervention group (% of group total)	Control group (% of group total)
Foot	8 (14.8%)	2 (20.0%)	6 (13.6%)
Ankle	7 (13.0%)	0 (0.0%)	7 (15.9%)
Achilles	7 (13.0%)	1 (10.0%)	6 (13.6%)
Lower leg	7 (13.0%)	2 (20.0%)	5 (11.4%)
Knee	9 (16.7%)	3 (30.0%)	6 (13.6%)
Upper leg	8 (14.8%)	2 (20.0%)	7 (15.9%)
Lower back	7 (13.0%)	1 (10.0%)	6 (13.6%)
**Total**	**54 (100%)**	**10 (100%)**	**44 (100%)**

4.2. Sensitivity analysis primary outcome with different thresholds according to the as-treated approach

Table A2 provides the sensitivity analysis of injury (hazard) ratios with different thresholds for inclusion in the intervention group. Only the model with crude estimated is presented for each threshold. The mean hazard rate showed a trend for a decrease with a larger relative number of sessions ran with real-time feedback.

Importantly, each model also showed a significant effect of group (with a one-sided *p*-value) on the injury ratio of all injuries for the ≥70% and ≥75% thresholds, but not with the ≥50% threshold. The sensitivity analyses where only injuries that lasted >7 days were considered all showed no significant effect of group on the injury rate, although there was a similar trend for lower hazard and a decrease in hazard rate with higher thresholds.

Table A2. Sensitivity analysis injury rate with different thresholds for inclusion in the intervention group

Co-variate	≥50% (unadjusted model)		≥70% (unadjusted model)		≥75% (unadjusted model)	
	HR (95% CI)	*p*	HR (95% CI)	*p*	HR (95% CI)	*p*
**All injuries**						
Group*	0.61 (0.33-1.14)	**0.06** (0.12)	0.49 (0.23-1.04)	**0.03** (0.06)	0.47 (0.21-1.05)	**0.03** (0.07)
**>7 day injuries**						
Group*	0.84 (0.37-1.89)	**0.38** (0.76)	0.56 (0.19-1.64)	**0.15** (0.29)	0.46 (0.14-1.55)	**0.11** (0.21)

HR = Hazard ratio. HR values <1 indicate a lower injury (hazard) ratio. *Control group is reference; 95% confidence intervals represent two-sided confidence intervals. *P*-values also represent two-sided *p*-values. The **bold** *p*-value in brackets represents the one-sided *p*-value.

4.3. BREQ-2 results

25.2% (*n* = 53) of the sample completed both the pre-study and follow-up questionnaires on motivation and interest in continuing to use the ARION wearable and were therefore included in these analyses. There were no significant changes within each group for any of the BREQ-2 subscales, and the change score did also not differ significantly between the groups (Table A3). Individuals that dropped out for non-injury reasons did also not differ significantly from the individuals that did not dropout (Table A4).

Table A3. BREQ-2 results

Subscale	Intervention group			Control group			
	Pre	Post	Change	Pre	Post	Change	Difference in change
Amotivation	0.15 ± 0.49	0.50 ± 1.24	-0.37 ± 1.17; *p* = 0.26	0.65 ± 1.23	0.35 ± 0.95	0.29 ± 1.55; *p* = 0.23	-0.66 ± 0.41; *p* = 0.11
External regulation	0.25 ± 0.91	0.30 ± 1.13	-0.05 ± 0.41; *p* = 0.83	0.32 ± 1.30	0.56 ± 1.19	-0.24 ± 1.2; *p* = 0.17	0.18± 0.29; *p* = 0.53
Introjected regulation	2.95 ± 2.14	3.15 ± 2.58	-0.53 ± 1.90; *p* = 0.29	4.18 ± 2.22	4.26 ± 2.38	-0.09 ± 2.28; *p* = 0.82	-0.44 ± 0.62; *p* = 0.48
Identified regulation	13.9 ± 2.03	13.0 ± 3.72	0.26 ± 2.08; *p* = 0.61	13.2 ± 201	13.4 ± 2.03	-0.15 ± 2.23; *p* = 0.71	0.41 ± 0.62; p = 0.51
Intrinsic regulation	13.9 ± 1.77	12.8 ± 3.64	0.68 ± 1.77; *p* = 0.10	13.9 ± 2.09	13.9 ± 2.02	-0.03 ± 1.83; *p* = 0.36	0.71 ± 0.52; p = 0.18

*All *p*-values are uncorrected for multiple comparisons.

Table A4 reports the comparison of pre-study BREQ-2 outcomes between individuals that dropped out for non-injury reasons prior to 6 months and individuals that did not dropout prior to 6 months.

Table A4. Comparison of BREQ-2 outcomes between dropouts and non-dropouts

Subscale	Mean ± SD dropout (*n* = 13)	Mean ± SD no dropout (*n* = 36)	Difference (Mean ± SE)
Amotivation	0.00 ± 0.00	0.58 ± 1.08	0.58 ± 0.30; *p* = 0.06
External regulation	0.00 ± 0.00	0.44 ± 1.40	0.44 ± 0.39; *p* = 0.26
Introjected regulation	2.77 ± 1.88	3.92 ± 2.17	1.15 ± 0.68; *p* = 0.10
Identified regulation	13.0 ± 2.80	13.4 ± 1.73	0.44 ± 0.67; *p* = 0.51
Intrinsic regulation	12.9 ± 2.60	14.3 ± 1.61	1.36 ± 0.62; *p* = 0.03[#]

*All *p*-values are uncorrected for multiple comparisons.
[#] This comparison is non-significant (*p* = 0.17) after correcting for multiple comparisons.

4.4. Correlation change in performance and weekly training volume

Weekly training distance did not significantly correlate with the change in performance across both groups (r = 0.03, p = 0.85). After removal of one outlier, this correlation remained non-significant (r = -0.14, p = 0.38; Figure A5).

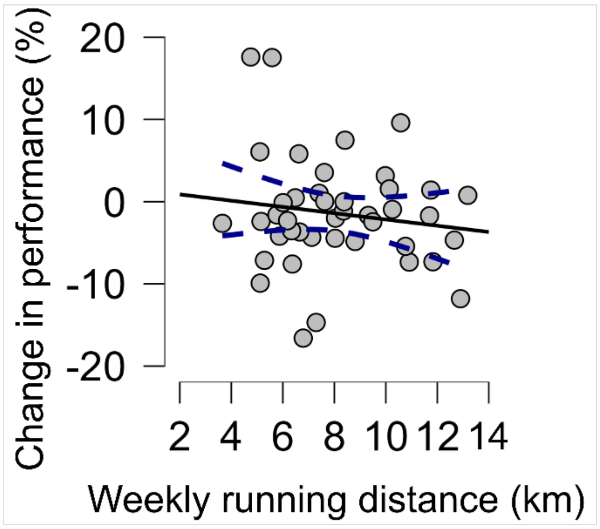

Figure A5. Correlation between weekly running distance and the change in self-reported personal best times. Negative changes in performance depict improvements. Dashed lines depict 95% confidence intervals.

Table A5. Median (interquartile range) percentage of sessions ran with each feedback option

**Outcome**	**% of sessions performed with each feedback modality relative to total number of feedback sessions**
Cadence	33.3 (21.9 – 50.9)
Footstrike index	25.0 (12.6 – 33.3)
Stable speed run	15.4 (6.39 – 26.8)
Speed play (interval)	5.88 (0.00 – 12.5)
Slow to fast speed	0.00 (0.00 – 16.9)

5. References

1. Van Hooren B, Willems P, Plasqui G, et al. The accuracy of commercially available wireless instrumented insoles (ARION) for measuring spatiotemporal running parameters. *Scandinavian Journal of Science and Medicine in Sports* 2023;33(9):1703-15. doi: 10.1111/sms.14424
2. Foster C, Porcari JP, Ault S, et al. Exercise Prescription When There Is No Exercise Test: The Talk Test. *Kinesiology* 2018;50(Supplement 1):33-48.
3. Van Hooren B, Goudsmit J, Restrepo J, et al. Real-time feedback by wearables in running: Current approaches, challenges and suggestions for improvements. *J Sports Sci* 2020;38(2):214-30. doi: 10.1080/02640414.2019.1690960 [published Online First: 20191203]
4. Markland D, Tobin V. A modification to the behavioural regulation in exercise questionnaire to include an assessment of amotivation. *Journal of Sport and Exercise Psychology* 2004;26(2):191-96.
